# Current Topics

**Published:** 1909-03-13

**Authors:** 


### Motoring Notes.

### CURRENT TOPICS.

### Lubrication of Springs.

There are many cars that worry their owners by squeaking on rough roads, and some that are offenders in this respect even on roads of very fair surface. The cause of the noise is frequently most puzzling to many drivers to locate when they know that the spring pins are properly lubricated. They, however, fail to recognise that the springs require lubrication between the plates or leaves of which they are composed.

If you have a car that squeaks mysteriously you should first satisfy yourself that it is not due to want of lubrication in any part of the mechanism which is constantly revolving, as this would be a very serious matter. Having satisfied yourself on this point, examine the springs and brake-rods. Oil all the bearings, and if they are provided with grease-lubricators remove these, fill them with soft grease, and before replacing clean out the hole in the spring pin with a wire. It may then be found that the squeak has disappeared. If not, attention should be paid to the plates or leaves of the springs. It is utterly impossible by any means to lubricate springs properly whilst the weight of the car is on them. The only way to manage it is to put a pair of jacks under the frame so that the weight is completely taken off the springs. In order to do this it will generally be found necessary to use a stout piece of wood a few inches wider than the frame and about three inches square, as the jacks have to be placed inside the frame to miss the springs. It will be found that the jacks will not be long enough to reach up to the wood, so it will be necessary to pack them up with some bricks or pieces of wood of a convenient size. Having carefully jacked up the car, the plates of the springs will come slightly apart, and can then be separated somewhat more by means of a strong screwdriver or similar instrument. Plenty of oil should then be poured in between each of the leaves, and a little grease afterwards pushed in with an old table-knife or with one of the flexible pallet-knives used by painters. The blade of the knife should be thickly greased and worked up and down between the leaves of the spring. This having been done, the car-body can be taken off the jacks and the weight taken up again by the springs, when it will be found that the squeaking has vanished.

The foregoing operation must be carried out both at the front and back of the car. Some people advocate the use of grease only for spring lubrication when the weight has been taken off them, but the advantages of the use of oil first are apparent, since it is obvious that it will find its way down between the leaves where the thinnest pallet-knife cannot be pushed without the grease being forced off the blade.

It will be found that the above treatment will not merely result in the stoppage of spring squeaks, but the springs themselves will work much more freely, and riding in the car will prove more comfortable over bad roads. I may say, perhaps, that it is not necessary to lubricate springs in this way unless they squeak, as some run almost indefinitely without any tendency to noise, but a large number will be all the better for the treatment, as apart from the unpleasantness of the squeaking the easy action of the springs is much improved by lubrication, and further, which is most important, rust is prevented.

It is, as a rule, evidence that springs are beginning to rust between the leaves when they commence to squeak, and even in the case of cars new from the factory it will, in some instances, be found that they run a very short distance before this trouble asserts itself. This is due to the care coachbuilders take in washing and cleaning springs before painting. They take such extra precautions to remove all dirt and grease that the cleaning material is apt to be forced between the leaves; and, as every particle of grease is removed from the outside of the springs, water gets between the leaves. Thus when the coachbuilder has finished with the springs there is very little grease left, and the more carefully he cleans them before painting the more effectually is every trace of lubricant removed. It must also be remembered that the more easy are the springs of a car, the more sensitive are they also to lack of lubrication, because the movement between the leaves of the spring, although small, is constant, and the more flexible the spring the greater the movement.

### Care of Brake Connections.

It is always wise when any defect in the brakes or steering mechanism is noticed to have it attended to without delay, since disaster may result from failure in the power of the former or defective action of the latter. In regard to the adjustment of brakes, provision is always made for shortening the tension rod so that the brake-pedal will not be as low down as it is possible to be pressed before the brake comes into full power.

The brake-pedal should always stand sufficiently high above the footboard to give ample clearance when the brake is as hard on as it can possibly be applied. In regard to the side-brake also, the hand-lever should not be much over half-way along the sector when the brake is nearly full on. Brake connections should be periodically examined, and any signs of failure remedied without delay. It is important to see that all nuts are tight, but as a rule in all first-class cars these are pinned, so that they cannot shake loose. With regard to the steering-joints, these must wear, since they are in constant use the whole time a car is running. The lost motion due to this will gradually increase, and if allowed to become too great adjustment is impossible, and replacement of parts becomes necessary. It may be taken as a rule that if the steering-wheel can be moved much over half an inch without deflection of the road wheels adjustment is necessary. It is, I hope, unnecessary for me to point out the evil of the very senseless habit some people have of turning the wheels of a car which is not in motion by means of the steering-wheel.

